# Formulation and Evaluation of pH-Modulated Amorphous Solid Dispersion-Based Orodispersible Tablets of Cefdinir

**DOI:** 10.3390/pharmaceutics16070866

**Published:** 2024-06-27

**Authors:** Yahya Alhamhoom, Thanusha Kumaraswamy, Avichal Kumar, Shivakumar Hagalavadi Nanjappa, Sanjana S. Prakash, Mohamed Rahamathulla, Kamal Y. Thajudeen, Mohammed Muqtader Ahmed, Thippeswamy Boreddy Shivanandappa

**Affiliations:** 1 Department of Pharmaceutics, College of Pharmacy, King Khalid University, Al Faraa, Abha 62223, Saudi Arabia; ysalhamhoom@kku.edu.sa (Y.A.); shmohamed@kku.edu.sa (M.R.); 2 Department of Pharmaceutics, KLE College of Pharmacy, Rajajinagar, Bengaluru 560010, India; thanushaswamy28@gmail.com (T.K.); avichalk0994@gmail.com (A.K.); sanjanasp307@gmail.com (S.S.P.); 3 Department of Pharmacognosy, College of Pharmacy, King Khalid University, Al Faraa, Abha 62223, Saudi Arabia; kthajudeen@kku.edu.sa; 4 Department of Pharmaceutics, College of Pharmacy, Prince Sattam Bin Abdul Aziz University, Al Kharj 11942, Saudi Arabia; muqtadernano@gmail.com; 5 Department of Biomedical Science, College of Pharmacy, Shaqra University, Al-Dawadmi Campus, Al-Dawadmi 11961, Saudi Arabia

**Keywords:** cefdinir, solid dispersion, pH-modulated, solubility, antimicrobial activity

## Abstract

Cefdinir (CEF) is a semi-synthetic third-generation broad-spectrum oral cephalosporin that exhibits poor solubility at lower pH values. Considering this, pH-modulated CEF solid dispersions (ASDs) were produced by solvent evaporation method employing various hydrophilic carriers and alkalizers. Among different carriers, ASDs produced using PEG 6000 with meglumine as alkalizer were found to significantly increase (*p* < 0.005) the drug solubility (4.50 ± 0.32 mg/mL) in pH 1.2. Fourier transform infrared spectrophotometry confirmed chemical integrity of CEF while differential scanning calorimetry (DSC) and X-ray diffractometry (XRD) indicated CEF was reduced to an amorphous state in ASD8. Antimicrobial assay performed by well diffusion method against *Staphylococcus aureus* (MTCC96) and *Escherichia coli* (MTCC118) demonstrated significantly superior (*p* < 0.001) efficacy of CEFSD compared to CEF. The porous orodispersible tablets (ODTs) of ASD8 (batch F5) were developed by incorporating ammonium bicarbonate as a subliming agent by direct compression, followed by vacuum drying displayed quick disintegration (27.11 ± 1.96 s) that met compendial norms and near-complete dissolution (93.85 ± 1.27%) in 30 min. The ODTs of ASD8 appear to be a promising platform to mitigate the pH-dependent solubility and dissolution issues associated with CEF in challenging physiological pH conditions prevalent in stomach. Thus, ODTs of ASD8 are likely to effectively manage various infections and avoid development of drug-resistant strains, thereby improving the curing rates.

## 1. Introduction

Lower respiratory tract infections, encompassing conditions such as bronchitis and pneumonia, are widely recognized for their impact on the respiratory system, often leading to significant respiratory distress and complications. Pneumonia is an infection of the lungs that primarily affects the alveolar space. It is characterized by inflammation and consolidation of the affected lung tissue. When microorganisms such as bacteria, viruses, or fungi invade the alveolar space and trigger an inflammatory response, it can cause pneumonia. This immune response results in symptoms such as cough, fever, chest pain, and difficulty breathing [1]. The more distal the infection in the lungs, the greater the chances of higher severity of the disease. In spite of advances in clinical therapy, pneumonia continues to be associated with high morbidity and mortality worldwide [2,3]. Pneumonia is the eighth leading cause of death and first among infectious diseases. Community-acquired pneumonia involves a high rate of intensive care admissions and treatment costs. The mortality rate is as high as 23% for patients admitted to the intensive care unit. The estimated worldwide occurrence of community-acquired pneumonia was found to range between 1.5 to 14 cases per 1000 person-years, which is affected by geography, season, and population characteristics [4]. The mortality is known to increase to an extent of 40% with old age and comorbidities [5]. For many years, the backbone of treatment for pneumonia has been the β-lactam class of antibiotics, including the third and fourth-generation cephalosporins and β-lactam/β-lactamase inhibitor combinations like piperacillin/tazobactam. Unfortunately, the ongoing spread of extended-spectrum β-lactamases (ESBLs) and carbapenemases such as *Klebsiella pneumonia* carbapenemase (KPC) has begun to limit clinical effectiveness of β-lactam agents over the last decade [6].

Cefdinir (CEF) is a semi-synthetic third-generation broad-spectrum oral cephalosporin antibiotic effectively active against Gram-positive and Gram-negative bacteria. It is used in the treatment of acute chronic bronchitis, pneumonia, rhinosinusitis, and pharyngitis. It is categorized under class IV of the biopharmaceutical classification scheme (BCS) as it exhibits solubility and permeability issues [7]. CEF, being a weakly acid drug, is known to exhibit poor solubility in acidic pH below 4.0. The pH-dependent solubility results in low dissolution rate that limits the absorption and its oral bioavailability of CEF (16–21%) [8]. In addition, the drug is known to possess a short half-life (1.7 h) due to rapid systemic clearance [9]. Poor bioavailability would in turn substantially reduce the antimicrobial activity, which eventually would result in the development of drug-resistant strains, affecting the curing rates and the therapeutic outcomes [10]. Therefore, attempts have been made to improve the solubility and dissolution of CEF employing several approaches like cyclodextrin complexation [10,11] and nanosizing [8]. Most importantly, studies undertaken of late have indicated that CEF would be absorbed from the duodenum and jejunum segment of the intestine [12]. In this context, there is a strong need to improve the solubility and dissolution of CEF in acidic conditions of the stomach. Considering pH-dependent solubility and the site-specific absorption of drug, the present study aims to mitigate the effect of physiological pH prevalent in the stomach by formulating pH-modulated amorphous solid dispersions of CEF (ASD) using hydrophilic polymers and alkalizing agents. In this context, alkalizers, namely sodium bicarbonate and meglumine, are employed in the present study to modulate the microenvironment pH, and therefore improve the dissolution of CEF. In immediate release dosage forms, alkalizers have the potential to modulate the microenvironment pH, minimizing drug precipitation and thereby improving the dissolution [13]. Subsequently, the developed ASD is incorporated into orodispersible tablets (ODT). The ASD-loaded ODT is an attractive platform to deliver CEF in a soluble form in the stomach. Moreover, the good palatability of CEF further prompted the development of ODTs [14]. Delivering the drug in soluble form proximal to the absorption window is a practically feasible approach to improve the absorption, and therefore the oral bioavailability, of CEF. The development of ODTs of ASD is novel and unique, as no such formulations of CEF has been reported prior to this study as far as our knowledge goes.

## 2. Materials and Methods

### 2.1. Materials

Cefdinir was obtained from Covalent Laboratory Pvt. Ltd., Hyderabad, India. Pearlitol^®^400 DC and Croscarmellose Sodium samples were obtained from Roquette (I) Pvt. Ltd. Mumbai, India. Avicel^®^ PH 102 samples were obtained from FMC (I) Pvt. Ltd. while crospovidone and Soluplus^®^ samples were obtained from BASF. Meglumine was purchased from Yarrow Chemicals, Mumbai, India. PEG 6000 and rest of the chemicals and reagents of analytical grade were acquired from S.D Fine Chemicals, Mumbai, India.

### 2.2. Phase Solubility Study

The phase solubility studies were carried out in a triplicate manner adopting the method reported by Higuchi and Connors [15]. Excess CEF was added to different concentrations of aqueous solutions of carriers such as PEG-6000, Soluplus^®^, and PVP K30 (0%, 3%, 6%, 9%, 12%, 15% *w*/*v*). The glass vials were intermittently agitated for 48 h on a mini rotary shaker (Remi Electrotechnik Limited, Mumbai, India). After equilibration, the supernatant liquid was filtered through a 0.45 µm filter paper. The absorbance was measured at 280 nm and assayed for the amount of CEF dissolved in the aqueous solutions. The calculation of the stability constant (Ks) was done using the slope and intercept of the phase solubility graph, as given in Equation (1).

(1) $$\mathrm{Ks}=\frac{Slope}{Intercept(1-slope)}$$

### 2.3. Preparation of Solid Dispersion

**Figure 1:**
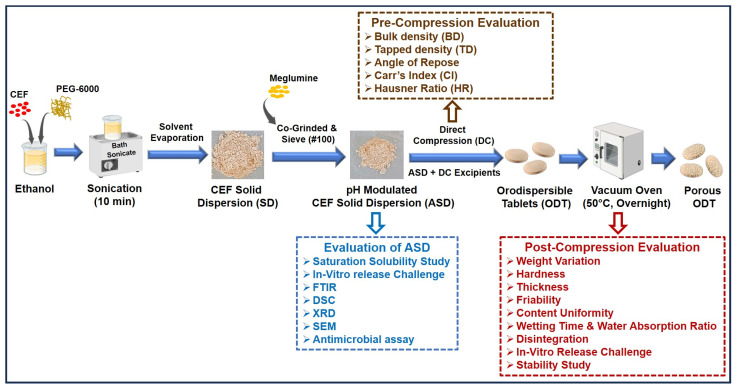
Schematic illustration of formulation development of pH-modulated solid dispersions and porous orodispersible tablets of Cefdinir with evaluation parameters.

Phase solubility studies were employed to screen a set of carriers that included Soluplus^®^, PEG 6000, and PVP K30. The selection of PVP K30 and PEG 6000 as carriers for preparing CEF solid dispersions (SDs) was based on the fact that they were found to display better solubility compared to Soluplus^®^. The SDs were produced employing PVP K30 and PEG 6000 as carriers at drug:carrier ratios of 1:1 and 2:1 by the solvent evaporation method [9]. The drug and the carrier were co-dissolved in a sufficient amount of ethanol and sonicated (GT sonic, Meizhou, China) for 10 min to obtain a clear solution. The solutions obtained were evaporated at 50 °C in a vacuum dryer (Servewell instruments Pvt. Ltd., Bengaluru, India) to obtain the solid dispersions (SD1–SD4). Finally, to the obtained SDs, alkalizers such as meglumine/sodium bicarbonate were added and co-grinded, passed through a sieve no #100, and stored for further characterization (ASD1–ASD8) as indicated in Figure 1. The compositions of different batches of SD and ASD are indicated in Table 1.

### 2.4. Characterization of Solid Dispersion

#### 2.4.1. Saturation Solubility Study

The saturation solubility of the CEF and SDs were performed by shake flask technique [15]. The excess amounts of samples were added to 5 mL of buffer of pH 1.2. The solutions were equilibrated at a temperature of 25 °C for 24 h with intermittent shaking. Subsequently, the dispersions were filtered using a filter paper of 0.45 µm pore size. The absorbance of the filtrate was measured at 280 nm after suitable dilution in a UV spectrophotometer (UV-1900i, Shimadzu Corporation, Tokyo, Japan) to determine the saturation solubility of the samples. The results were expressed as mean ± standard deviation (S.D) of three determinations.

#### 2.4.2. In Vitro Release Challenge Studies

The in vitro release results of SD and ASD were challenged in 900 mL at pH 1.2 in a USP dissolution test type II apparatus (TDT-08L-Electrolab, Mumbai, India) [11]. The medium was maintained at 37 ± 0.5 °C with a stirring speed of 50 rpm. Samples of 5 mL were withdrawn at 5, 10, 15, 20, 25, and 30 min and replaced with fresh buffer maintained at the same temperature. The absorbance was measured at 280 nm and assayed spectrophotometrically to determine the amount of drug dissolved at different time points after appropriate dilution. The results of the studies were expressed as mean ± S.D of three determinations.

#### 2.4.3. Fourier Transform Infrared Spectroscopy

The Fourier transform infrared (FTIR) spectrometry was used to assess the compatibility between CEF, excipients used, physical mixture, SD, and ASD [11]. The samples were mixed with potassium bromide and placed into a diffuse reflectance sampler for exposure to IR radiation. The samples were scanned in the range of 400–4000 cm^−1^ with a FTIR spectrophotometer (Jasco 450 Plus, ECC 450, Easton, MD, USA) to record the IR spectra.

#### 2.4.4. Differential Scanning Calorimetric Analysis

Differential scanning calorimetry (DSC) is a major thermoanalytical technique used to characterize the solid state of CEF in the SD and ASD [16]. DSC analysis of CEF, excipients, physical mixture, SDs, and ASDs were performed in a differential scanning calorimeter (DSC-60-Shimadzu, Tokyo, Japan). The samples were sealed in an aluminum crucible and analyzed at a heating rate of 10 °C/min in the temperature range of 25–250 °C with purging of nitrogen (50 mL/min). The degree of crystallinity (Xc) of the samples was calculated by Equation (2):

(2) $$\mathrm{Xc}=\frac{∆Hm}{(1-w)\times {∆H}^{0}m}\times 100$$

where ∆Hm is the measured heat of fusion, ∆H⁰m is the heat of fusion of 100% crystalline CEF, and w is the weight fraction of the sample in the carrier matrix.

#### 2.4.5. Powder X-ray Diffraction Study

XRD has been employed along with DSC to study the solid state of the drug present in the samples [10]. The diffraction studies were carried out in a powder X-ray diffractometer (D8 Advance, Bruker, Karlsruhe, Germany). X-ray diffractograms of CEF, excipients, physical mixture, and SDs were recorded individually at 40 kV voltage and current 30 mA at a scanning speed of 0.5 s per step. The data were acquired in 2θ values ranging from 10 to 50°. The degree of crystallinity (DC) was calculated according to the Equation (3):

(3) $$\mathrm{RDC}=\frac{I_{sample}}{I_{refer}}\times 100$$

where I_sample_ is the peak height of the sample that displayed the highest intensity and I_refer_ is the peak height of CEF at the same 2θ value.

#### 2.4.6. Scanning Electron Microscopy

Scanning electron microscopy (SEM) was employed to assess the morphology and surface topography of CEF and ASD [11]. The samples to be observed were mounted on an SEM sample stab using a double side sticking tape and gold coated (~200 nm) under reduced pressure (0.0133 Pa) for 5 min using an ion sputtering device at 10 kV energy using an EHT detector. The SEM photographs of the appropriate resolution were acquired using SEM (Tescan Vega 3, Brno, Czech Republic).

#### 2.4.7. Antimicrobial Assay

In vitro antimicrobial assay of the ASD was performed by well diffusion method against Staphylococcus aureus (MTCC96) and *E. coli* (MTCC118) [10]. The organisms were grown in Brain Heart Infusion (BHI) media for 24 h at 37 °C. The BHI media (1.5% *w*/*v* agar) pre-inoculated with the bacteria (1% *v*/*v*), was pour plated into a sterile petri dish and allowed to solidify. Wells of 4 mm were bored using a sterile cork borer and inoculated with the samples containing CEF (10 μg/mL). The plates were chilled for 30 min at 4 °C to ensure proper diffusion. Afterward, the plates were placed in an incubator set at 37 °C for a duration of 24 h. During this time, careful observations were made to determine the zone of inhibition (mm in diameter).

### 2.5. Formulation of Porous Orodispersible Tablets

The porous ODTs were developed by the direct compression of the optimized batch of ASDs while those produced with CEF served as a reference. The process of production of the porous ODTs is conceptualized in Figure 1. The compositions of the total eight batches of ODT produced by direct compression are depicted Table 2. The ingredients were thoroughly mixed with other excipients in a polybag and then passed through a sieve no #44 and evaluated for granular properties before direct compression. The lubricated blend was directly compressed into round tablets weighing 150 mg using flat-faced bevel-edge punches in rotary tablet press (Model RSB-4, RIMEKMINI PRESS, Ahmedabad, India). About 50 tablets were compressed for each batch using B tooling of 8.75 mm diameter to a hardness ranging between 3–4 Kg. The tablets produced of batch F1, F3, F4, and F5 that contained ammonium bicarbonate as subliming agent were finally dried at 50 °C overnight in a vacuum dryer [17].

### 2.6. Evaluation of Pre-Compression Properties

The granular properties of pre-compressional blend including bulk density (BD), tapped density (TD), and angle of repose were determined. The Carr’s index (CI) and Hausner ratio (HR) were calculated using Equations (4) and (5), respectively [18].

(4) $$\mathrm{CI}=\frac{TD-BD}{TD}\times 100$$

(5) $$\mathrm{HR}=\frac{TD}{BD}\times 100$$

### 2.7. Evaluation of Rapid Orodispersible Tablets

The tablets of different batches were evaluated for various post-compression parameters to assess whether they met the compendial standards listed in Indian Pharmacopeia [19].

#### 2.7.1. Weight Variation

The weight of twenty tablets from each batch was individually weighed using an electronic balance (Shimadzu BL-220H, Kyoto, Japan) and the average weight was calculated. Each tablet’s weight was then determined and compared to the average value [19]. The results of the weight uniformity were expressed as mean ± S.D.

#### 2.7.2. Hardness

The hardness of 10 randomly selected tablets from each batch of ODTs were measured using a Pfizer hardness tester (Lab Junction, LJ-200, Mumbai, India) [19]. The results were expressed as mean ± S.D.

#### 2.7.3. Thickness

Three tablets were chosen randomly from each batch of the ODTs. The thickness of 10 tablets was determined using a digital vernier caliper (Mitotoyo Corporation, Tokyo, Japan) [19]. The results were expressed as mean ± S.D.

#### 2.7.4. Friability

Compressed tablets weighing 6.5 g were used for the evaluation of friability. The tablets were subjected to a test that spanned 4 min in a friabilator (Model EF-20, Electrolab, Mumbai, India) at 25 rpm [19]. The loss in the weight of tablets due to attrition or breakage was recorded to determine the percentage of friability (%F), calculated based on Equation (6), and the results were expressed as mean ± S.D.

(6) $$\%F=\frac{W-W_{0}}{W_{0}}\times 100$$

where W = Initial weight of the tablet and W_0_ = Weight of tablet after the test.

#### 2.7.5. Content Uniformity

The drug content was evaluated by the triturating ten individual tablets in order to determine the content uniformity. The pre-determined weight of powder was dissolved in phosphate buffer pH 7.4 in a volumetric flask. The absorbance was measured at 280 nm and assayed using the calibration curve constructed in the same media to determine the amount of drug present in the ODTs. The experiments were performed in triplicate and the results were expressed as mean ± S.D.

#### 2.7.6. Wetting Time and Water Absorption Test

A squared piece of tissue paper was rolled twice and placed in the small petri plate with a diameter of 5.5 cm. To the plate, about 10 mL of distilled water was added [20]. Subsequently, a tablet was placed on the moist tissue paper and the total wetting time of each ODT was noted. The water absorption ratio (R) was calculated after reweighing the wetted tablet using Equation (7). The experiments were performed in triplicate and the results were expressed as mean ± S.D.

(7) $$R=[(W_{a}-W_{b})/W_{b}]\times 100$$

where W_a_ and W_b_ denote the weight of the tablet after and before the study was conducted.

#### 2.7.7. Disintegration Test

The disintegration time of tablets was determined using USP disintegration test apparatus (Electrolab ED-2L, Mumbai, India). One tablet was dropped into each of the six tubes of the apparatus containing water as the media, following which a disc was introduced to each tube. The buffer temperature was maintained at 37 ± 2.0 °C [19]. The time taken for the tablet to completely disintegrate was recorded and the mean standard deviation of six determinations was reported.

#### 2.7.8. In Vitro Release Challenge Studies

The in vitro release results of ASD-loaded ODTs were challenged in 900 mL of pH 1.2 in a USP dissolution test type II apparatus (Electrolab TDT-08L, Mumbai, India). The test was carried out in 900 mL of pH 1.2 using acidic buffer as the medium, maintained at a temperature of 37 ± 0.5 °C and stirring speed of 50 rpm [11]. About 5 mL of samples were withdrawn at 5, 10, 15, 20, 25, and 30 min and replaced with fresh pH 1.2 acidic buffer maintained at the same temperature. The absorbance was measured at 280 nm and assayed for the amount of drug dissolved at different time points. A graph of amount of drug release was plotted against time to obtain the dissolution profile. The pH of the dissolution media was measured after completion of the studies. The similarity factor (f_2_) and dissimilarity factor (f_1_) were used to compute and compare the dissolution profiles of the two formulations using Equation (8) and Equation (9), respectively [21]:

(8) $$f_{1=}\frac{\left\{\left\{\sum_{t=1}^{n}\left|Rt-Tt\right|\right\}\right\}}{\sum_{t=1}^{n}Rt}\times 100$$

(9) $$f_{2}=50\times \log\left[{\left\{1+\frac{1}{n}\sum_{r=1}^{n}wt\left(Rt-Tt\right)\right\}}^{-0.5}\times 100\right]$$

where n is the number of observations, Rt is the average percentage drug dissolved for reference formulation and Tt is the average percentage drug dissolved for test formulation. Wt is the weight factor that is usually considered as 1.

#### 2.7.9. Stability Studies

The selected formulation was further subjected to long-term stability studies for up to 3 months at temperature conditions 25 ± 2 °C and 40 ± 2 °C with relative humidity (RH) 60 ± 5% and 75 ± 5%, respectively, for a period of 3 months [15]. The tablets were examined at the conclusion of the study for critical parameters like weight variation, friability, hardness, disintegration time, drug content, and drug release profile of the drug. The data generated were compared with those recorded before subjecting the tablets to the studies.

### 2.8. Statistical Analysis

The results obtained were statistically analyzed using unpaired Student’s *t* test or one-way ANOVA. A *p*-value of less than 0.05 was considered to be statistically significant. The data generated were expressed as mean ± S.D. The values of S.D are represented as error bars in the graphs.

## 3. Results

### 3.1. pH Solubility Profiling

**Figure 2:**
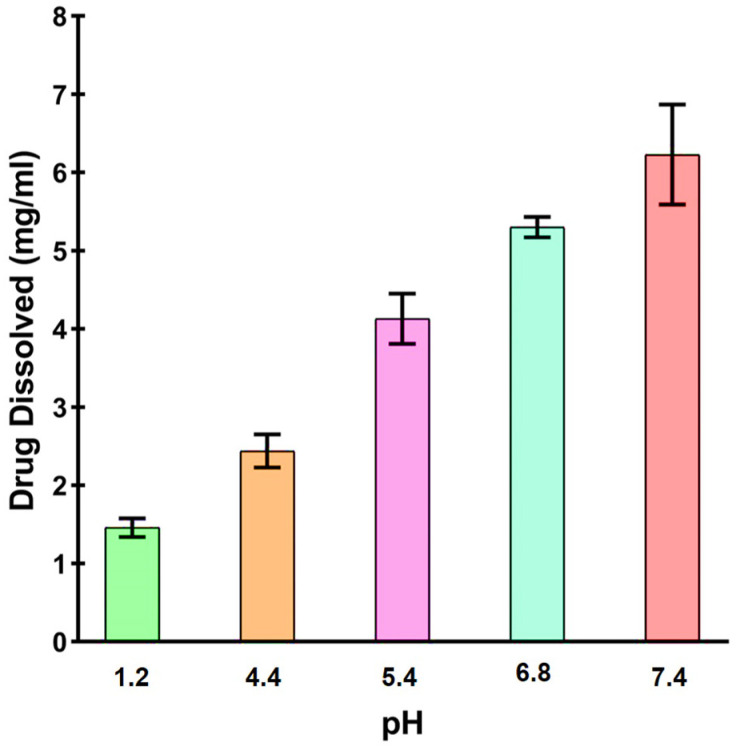
pH-dependent solubility profiles of CEF at varying pH.

The pH-dependent solubility of CEF was clearly evident as it displayed the least solubility in pH 1.2, while the highest solubility was noted at pH 7.4, as indicated in Figure 2. The solubility at pH 1.2, 4.4, 5.4, 6.8, and 7.4 was found to be 1.45 ± 0.12, 2.43 ± 0.21, 4.15 ± 0.32, 5.2 ± 0.13, and 6.18 ± 0.64 mg/mL, respectively.

### 3.2. Phase Solubility Study

**Figure 3:**
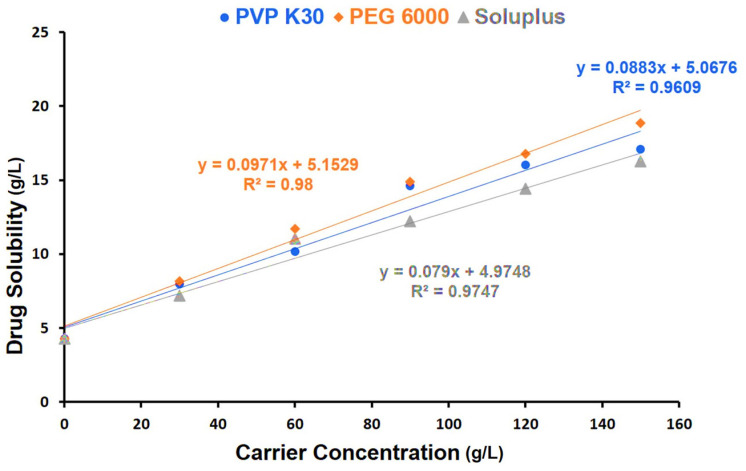
Phase solubility profiles of CEF with hydrophilic carriers (PVP K30, PEG 6000, and Soluplus).

The apparent solubility of the CEF in water obtained directly from the Y-axis intercept was found to be 4.95 ± 0.18 mg/mL, as indicated in Figure 3. The linearity of the curve indicated the formation of amorphous solid dispersion with hydrophilic polymers. Thus, the phase solubility diagram can be classified as A_L_ type. The stability constants computed from the intercept and slope are tabulated in Table 3. The studies indicated that PEG 6000 was found to be more effective compared to other hydrophilic polymers in improving the solubility of CEF.

### 3.3. Formulation of Solid Dispersion of Cefdinir

A clear solution was obtained when the drug and the carrier were co-dissolved in ethanol. The solvent evaporation technique employed would enable uniform dispersion of CEF in the carrier. On rapid evaporation of ethanol by vacuum drying, a residue of CEF solid dispersion was obtained, resulting in formation of SD [9]. SDs are generally formed when the drug is dispersed in molecular state in amorphous hydrophilic polymer matrix. SDs thus obtained were co-grinded with sodium bicarbonate/meglumine to finally produce the ASDs.

### 3.4. Characterization of Solid Dispersion of Cefdinir

#### 3.4.1. Saturation Solubility Studies of Solid Dispersion

**Figure 4:**
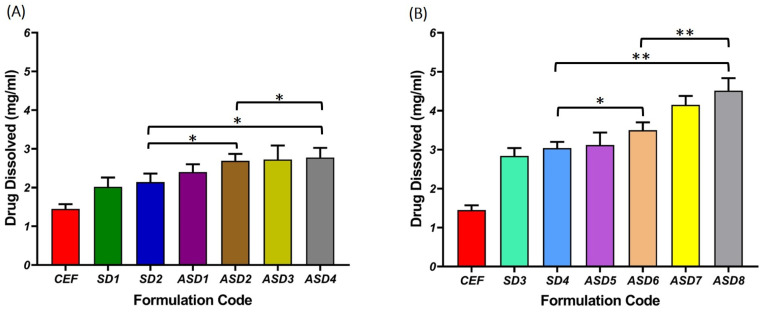
Saturation solubility of CEF from solid dispersions (SD) and pH-modulated solid dispersions (ASDs) of (**A**) PVP K30 and (**B**) PEG 6000. Level of significance based on the ANOVA: * *p* < 0.05; ** *p* < 0.01.

The saturation solubility indicating the maximum solubility of CEF in pH 1.2 at 25 °C from SDs and ASDs are depicted in Figure 4A and Figure 4B, respectively. The media (pH 1.2) employed to determine the solubility was found to be quite discriminatory in identifying the right hydrophilic carrier for development of CEFSD. Generally, the saturation solubility of CEF composed of SDs of PEG 6000 was found to be significantly higher (*p* < 0.05) compared to those made of PVP K30. Likewise, ASDs displayed a better solubility compared to SDs composed of the same carriers. In addition, the saturation solubility of CEF was found to be significantly higher (*p* < 0.01) for ASDs of PEG 6000 containing meglumine compared to those made of bicarbonate. Overall, ASD8 composed of PEG 6000 at drug-to-PEG 6000 ratio of 1:1 with meglumine as alkalizer displayed significantly better (*p* < 0.05) solubility compared to other ASDs.

#### 3.4.2. In Vitro Release Challenge Study of Solid Dispersion of Cefdinir

**Figure 5:**
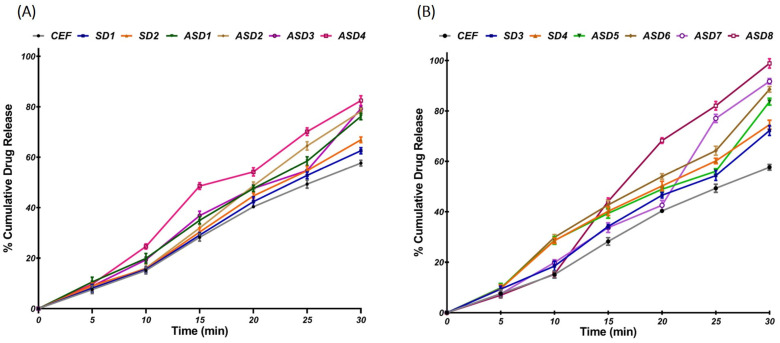
In vitro release profiles comparison of CEF from solid dispersions (SD) and pH-modulated solid dispersions (ASDs) composed of (**A**) PVP K30 and (**B**) PEG 6000.

CEF was found to display dissolution of 57.62 ± 4.21% in the stipulated period of 30 min in pH 1.2. The drug release from solid dispersions generally depended on the type of carrier, drug-to-carrier ratio and the alkalizer used. Generally, the drug release was found to be significantly higher in SDs and ASDs composed of PEG 6000 compared to those made of PVP K30. Likewise, it was observed that ASDs displayed higher drug release compared to the SDs. Similarly, the drug release was found to be better when meglumine was used as alkalizer compared to bicarbonate. ASD8, composed of PEG 6000 as carrier and meglumine as alkalizer, proved to be the most promising ASD with a release of 97.34 ± 1.68% in 30 min that was significantly higher (*p* < 0.01) than the release observed with other ASDs (Figure 5).

#### 3.4.3. Fourier Transform Infrared Analysis

**Figure 6:**
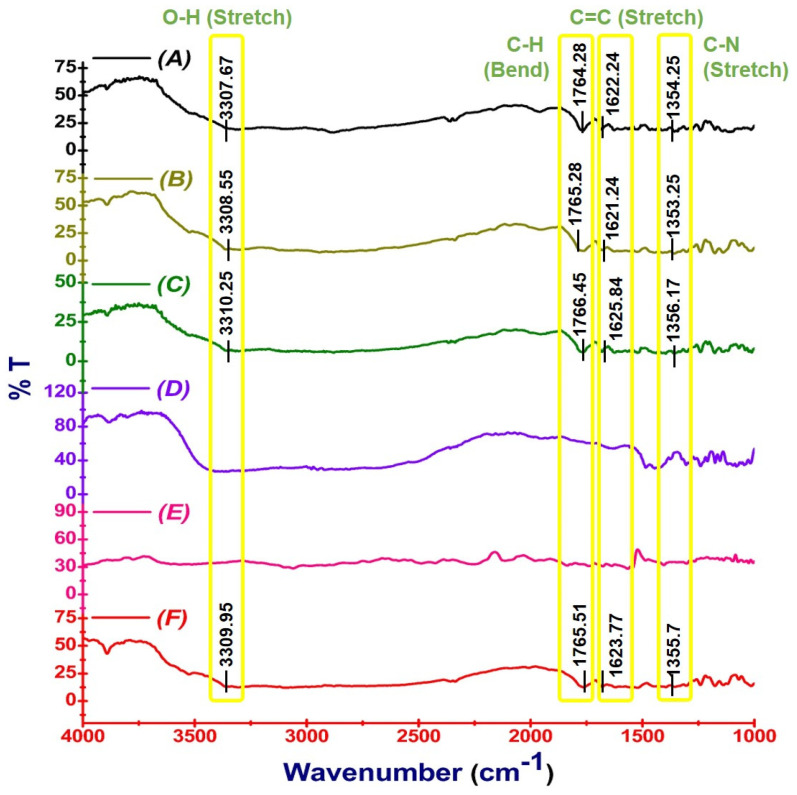
FTIR vibrational band of ASD8 (**A**), SD4 (**B**), physical mixture (**C**), meglumine (**D**), PEG 6000 (**E**), and CEF (**F**).

CEF was found to display characteristic peaks at 1765.51, 1623.77, and 1355.7 cm^−1^ (Figure 6F). All the prominent peaks of CEF were present in the physical mixture, ruling out the chemical interaction between drug and excipients in the physical mixture (Figure 6C). Likewise, the drug characteristic peaks were clearly evident in SD4 (Figure 6B) and ASD8 (Figure 6A), indicating the chemical integrity of CEF in the solid dispersions.

#### 3.4.4. Differential Scanning Calorimetry

**Figure 7:**
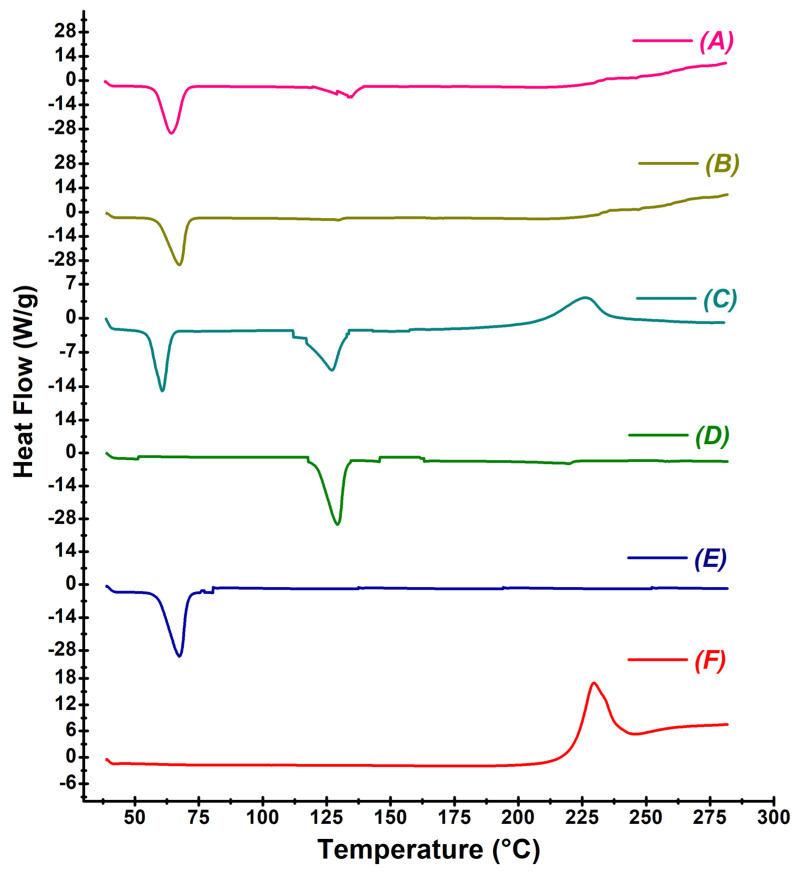
Differential scanning calorimetric thermograms of ASD8 (**A**), SD4 (**B**), physical mixture (**C**), meglumine (**D**), PEG 6000 (**E**), and CEF (**F**).

The DSC thermograph of CEF was observed at 229.56 °C with an enthalpy of fusion (∆Hf) value of 239.35 J/g, which confirms its crystallinity nature in CEF as indicated in Figure 7F. Meglumine displayed a characteristic exothermic peak at 130 °C (Figure 7D) while PEG 6000 exhibited an exothermic peak at 63 °C (Figure 7E) that corresponded to the respective melting points. The sharp endothermic peak of CEF appeared in the physical mixture (Figure 7C) at 226.19 °C, though at low intensity, indicating the crystallinity of the drug still persists. Further, the drug characteristic peak nearly disappeared in ASD8 (Figure 7A) and SD4 (Figure 7B) with a heat of fusion 2.75 J/g and 2.82 J/g, respectively, indicating a drop in crystallinity to ~2% of CEF that signified considerable amorphization of the CEF in SD4 and ASD8.

#### 3.4.5. X-ray Powder Diffraction

**Figure 8:**
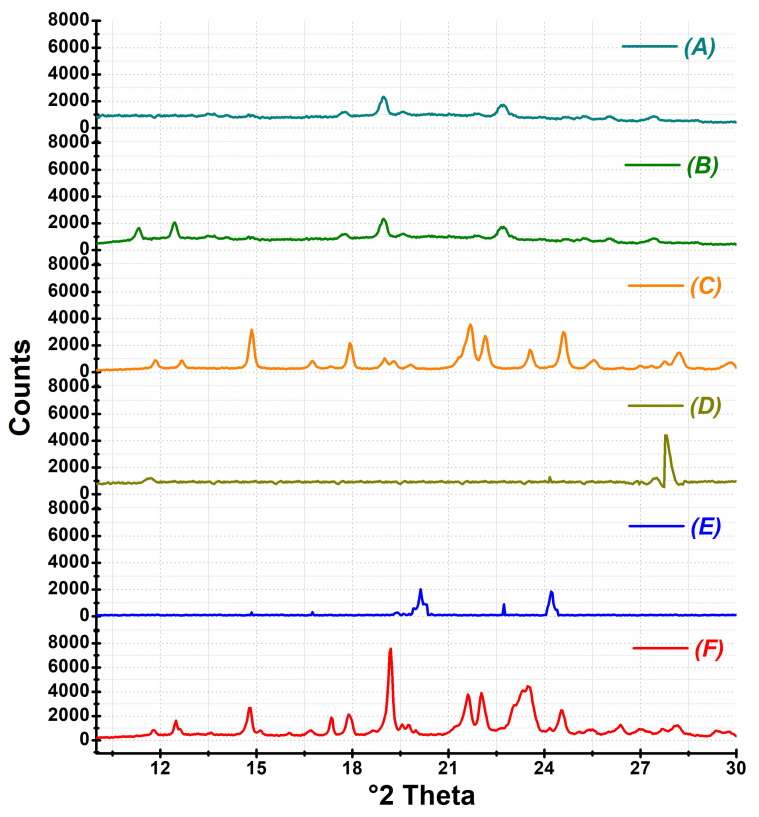
X-ray diffractograms of ASD8 (**A**), SD4 (**B**), physical mixture (**C**), meglumine (**D**), PEG 6000 (**E**), and CEF (**F**).

The X-ray diffractogram of CEF was found to display 10 characteristic peaks at 2θ values of 14.81°, 17.88°, 19.04°, 19.16°, 21.52°, 21.64°, 22.15°, 23.54°, 24.56°, and 25° (Figure 8F). The diffractogram of physical mixture displayed the CEF characteristic low intensity peaks at 2θ values of 14.89°, 17.91°, 19°, 21.60°, 21.64°, 21.68°, 22.11°, and 23°, indicating that CEF was existing in semi-crystalline state (Figure 8C). However, characteristic CEF peaks including the reference peak ~19° were considerably reduced in intensity in the PXRD spectra of ASD8 (Figure 8A) and SD4 (Figure 8B), indicating considerable amorphization of CEF.

#### 3.4.6. Scanning Electron Microscopy

**Figure 9:**
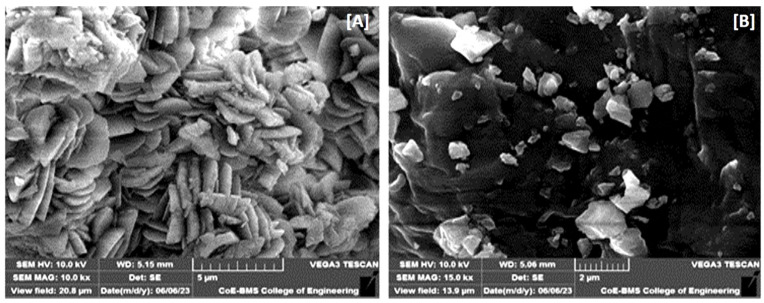
Photomicrographs of drug under magnification of 10,000× (**A**) and ASD8 under magnification of 15,000× (**B**).

The photomicrographs at a magnification of 10,000× indicate that the CEF particles displayed a large particle size, which is likely to hamper the solubility and dissolution rate (Figure 9A). On the contrary, photomicrographs of solid dispersion revealed that the particles of ASD8 were considerably reduced in size even when observed under a higher magnification of 15,000× (Figure 9B).

#### 3.4.7. Antimicrobial Studies

**Figure 10:**
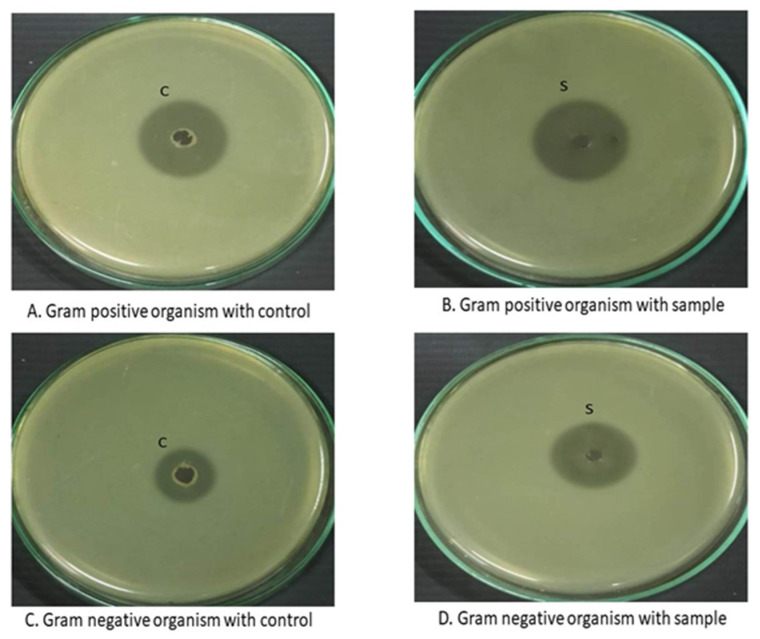
Zones of inhibition of CEF used as control against Gram-positive (**A**) and Gram-negative (**C**) organisms. Zones of inhibition of samples (ASDs) against Gram-positive (**B**) and Gram-negative (**D**) organisms.

The results of the antimicrobial studies of CEF and ASD8 against Gram-positive (*S. aureus*) and Gram-negative (*E. coli*) species are captured in Figure 10. The zone of inhibition (ZOI) for CEF that was employed as a reference measured 22 ± 1 and 14 ± 1 mm in diameter for *S. aureus* and *E. coli*, respectively. On the other hand, the zone of inhibition for ASD8 was found to be 26 ± 1 and 18 ± 1 mm in diameter for *S. aureus* and *E. coli*, respectively. Unpaired Student’s *t* test indicated that ZOI for ASD8 was significantly higher (*p* < 0.001) against both organisms when compared with that produced by the CEF that was employed as control.

### 3.5. Formulation of the Orodispersible Tablets

#### Pre-Compression Tests

The results of the pre-compressional properties of the lubricated blend are captured in Table 4. The values of HR, CI, and angle of repose values for the blends produced with CEF and ASD8 indicated that the blend displayed good flow properties.

### 3.6. Evaluation of Orodispersible Tablets

#### 3.6.1. Post-Compression Parameters

**Figure 11:**
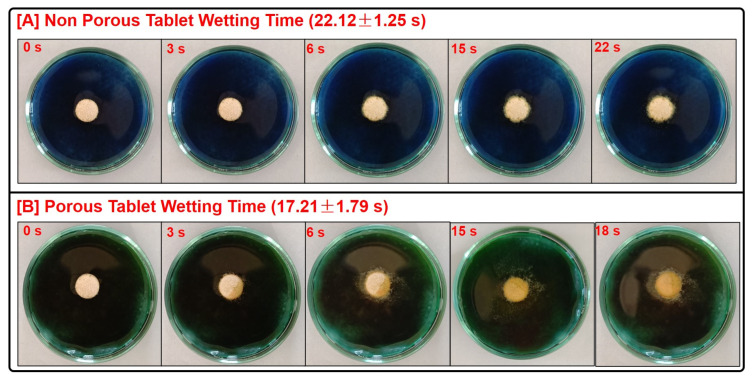
Pictorial representation of wetting time (s) of (**A**) non-porous orodispersible tablet of Batch F2 and (**B**) porous orodispersible tablet of Batch F5 at different time points.

The results of the post-compressional parameters are portrayed in Table 5. All the batches of tablets produced by direct compression were found to meet the pharmacopeial limits. The ODTs of batches F1, F3, F4, and F5 that were subjected to vacuum drying were found to meet the compendial limits as well. The hardness values for the prepared formulation ranged from 3.12 ± 0.28 kg to 3.50 ± 0.25 kg. Thickness of the prepared ODTs ranged from 2.99 ± 0.03 to 3.05 ± 0.06 mm, indicating the uniformity in fill weight. The friability for the prepared formulation ranged from 0.25 ± 0.04 to 0.29 ± 0.09, suggesting that the values were within the acceptable pharmacopeial limit of 1%. The assay for the tablets ranged from 88.32 ± 1.24 to 96.46 ± 084%, indicating uniformity in the content. The wetting time of all the tablet formulations was within the permissible limit of 1 min. The pictorial illustration of wetting time of ODTs is captured in Figure 11.

#### 3.6.2. In Vitro Release Challenge Studies

**Figure 12:**
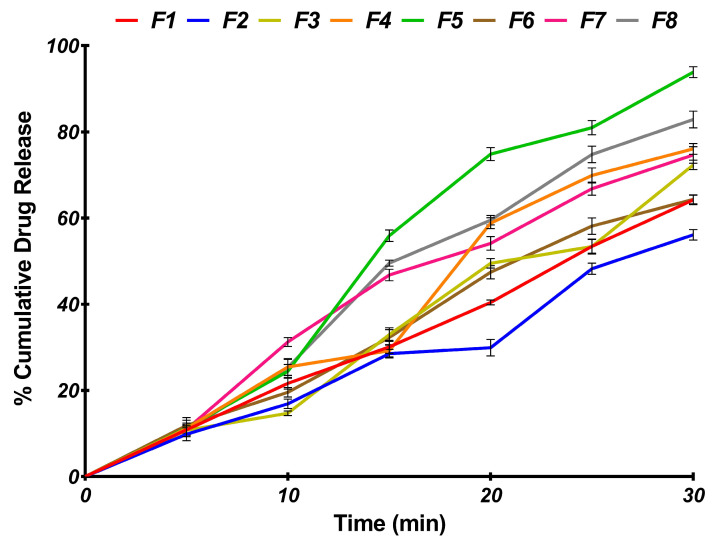
In vitro cumulative release profile of formulations F1–F8.

The drug release from the ODTs was found to depend on the solid state of the drug, tablet porosity, and DT. Thus, the porous tablets of batch F5 that exhibited the shortest DT and maximum drug release emerged as the optimized formulation of ODT, as indicated in Figure 12.

#### 3.6.3. Stability Studies

The tablets of batch F5 were evaluated for several physicochemical and post-compressional parameters following the stability studies. The results of the stability studies are captured in Table 6.

## 4. Discussion

CEF is a semi-synthetic third-generation broad-spectrum oral cephalosporin that is known to possess three ionizable groups that include a carboxylic acid group of cepham moiety having a pK_a_ of 1.8, an amine group of aminothiazole moiety with a pK_a_ of 3.35, and a = N-OH group of oxime moiety having a pKa of 9.9. Being a weak acid, CEF exhibits poor solubility at lower pH values that affects its oral bioavailability, and therefore the therapeutic efficacy. Considering this, the present investigation aimed to mitigate the negative effect of acidic pH in the stomach by formulating ASDs using hydrophilic polymers and alkalizers. The amorphous state of CEF in the hydrophilic carrier is likely to increase the aqueous solubility of the drug. The polymer drug interaction could be the result of formation of hydrogen bonds or hydrophobic interaction. Therefore, selection of the ideal carrier would be crucial to improve the solubility and dissolution in challenging physiological conditions. The study involved determination of pH solubility profile of CEF to assess the right combination of hydrophilic polymers and alkalizers to develop ASDs. CEF was found to possess poor solubility in acidic condition while it was readily soluble at alkaline pH. The solubility of CEF was found to significantly increase (*p* < 0.0005) by more than four-fold as the pH was increased from 1.2 to 7.4. This can be attributed to the fact that solubility of CEF is likely to increase substantially once the pH exceeds 3.35. A similar pH-dependent solubility of CEF that indicated increase in the solubility at pH values by nearly four-fold was reported earlier. The solubility generally increases once the pH of the media increases beyond the pK_a_ value of CEF, which is reported to be 1.9. The results observed in the present study were in close agreement with the report that indicated a solubility of 0.52 and 16.43 mg/mL at pH 2.5 and 8.0, respectively [7]. The poor solubility at low pH values prompted us to develop solid dispersions of CEF to resolve the associated solubility issues.

The solubility value (4.95 ± 0.18 mg/mL) deduced from the phase solubility studies performed in water was close to that reported earlier [7]. However, it was noted that the aqueous solubility of the CEF increased linearly as the carrier concentration was augmented. The increase in drug solubility is likely due to increased wettability of the solid dispersions formed by hydrophilic polymers [22]. A slope value of less than 1 with all the carriers investigated indicated the formation of SD at 1:1 ratio with the carriers [23]. The highest slope was noted for PEG 6000 (slope: 0.097) indicating considerable enhancement in drug solubility with increase in carrier concentration compared to that observed with PVP K90 (slope: 0.088) and Soluplus^®^ (slope: 0.079). At the highest carrier concentration PEG 6000 displayed nearly four-fold improvement in the drug solubility, indicating the possibility of formation of SD. CEF is reported to have a tendency to produce SD with hydrophilic polymers that was found to display improved solubility, dissolution, and bioavailability [7]. The stability constant (Ks) computed from the slope and the intercept of the phase solubility curve was found to be the highest for PEG 6000, as represented in Table 3 [23].

SD with PVP K30 and PEG 6000 as carriers were initially produced at a drug:carrier ratio of 1:1 and 2:1 by the solvent evaporation method (Table 1). Pre-formulation studies were undertaken to identify the right solvent that would be able to dissolve the CEF and the carrier. Ideally, the solvent used during formulation should not impact the stability of SDs during evaporation [22]. SDs are generally formed by rapidly evaporating the solvent from the drug–carrier solution [22]. Considering this, the alcoholic solutions of CEF and the polymers were subjected to rapid evaporation by vacuum drying. Later, considering the pH-dependent solubility of CEF, meglumine/sodium bicarbonate were employed as pH modulators in two different sets of ASDs in an attempt to improve the solubility of CEF in acidic pH. As both the alkalizers were found to be insoluble in ethyl alcohol, meglumine and sodium bicarbonate were co-grinded to obtain the solid dispersions. Meglumine and bicarbonate have been used as alkalizers to produce solid dispersions by co-grinding technique to modulate dissolution of drugs that display pH-dependent solubility [24]. The solubility of drugs that are pH dependent can be generally improved by incorporation of alkalizers that modulate the microenvironment pH of the dosage form. Alkalizers such as sodium bicarbonate and meglumine are known to modulate the microenvironment pH of the solid dispersion, thereby avoiding drug supersaturation. Alkalizers avoid potential drug precipitation in the microenvironment, thereby improving the dissolution rate of acidic drug [13]. The alkalizers used in the present study are generally regarded as safe (GRAS) at the concentration used in the present study, as per the Inactive Ingredient Guide portal of USFDA. Alkalizers in PEG 6000-based solid dispersions are reported to reduce the drug crystallinity, thereby improving the dissolution rate [25].

As there is a strong need to improve the solubility of CEF in pH 1.2, solubility studies were performed in the same media. The increase in saturation solubility of CEF can be attributed to the formation of SDs with hydrophilic carriers through hydrogen bonding or hydrophobic interaction. Amorphous SDs are normally formed when a drug is uniformly dispersed within a hydrophilic matrix material in an amorphous state. The amorphous or the molecular state of the CEF in SDs plays a crucial role in increasing their solubility as no energy is required to break the crystal lattice of crystalline drugs [22]. In case the drug solubility is less than the equilibrium solubility of the drug in the carrier, it would be dispersed in molecular state in the polymer matrix [26].

The saturation solubility of the ASD in pH 1.2 was found to depend on type and proportion of carrier used in addition to the type of alkalizer used. Generally, the increase in the carrier proportion enhanced the saturation solubility, as indicated in the phase solubility diagram (Figure 3). The drug solubility was reported to improve with an increase in proportions of hydrophilic polymer, due to higher chances of amorphization [26]. In the present study, drug solubility was found to be significantly increased (*p* < 0.05) for ASDs compared to SDs for the same carrier (Figure 4). Among the different batches, those produced using PEG 6000 as carrier and meglumine as pH modifier proved to be more effective in improving the drug solubility. Thus, ASD8 that was composed of drug-to-PEG 6000 in a ratio of 1:1 displayed the highest solubility of 4.51 ± 0.32 mg/mL. Alkalizers are known to play a critical role in modifying the microenvironment pH, avoiding possible drug precipitation and thereby demonstrating the ability to improve the solubility of drugs that display pH-dependent solubility [26]. The likely reason for the improvement in solubility in pH 1.2 is the ability of CEF to form ASD with PEG 6000. In addition, it has to be noted that meglumine has the ability of to modify the microenvironment pH, thereby improving the solubility of CEF as it is prone to be more soluble at pH exceeding 4.0. Modulation of microenvironment pH using alkalizers was employed as a practical strategy to improve the solubility of drugs that display pH-dependent solubility [24].

The results of in vitro release challenge studies performed for a period of 30 min in pH 1.2 correlated well with the saturation solubility data, as indicated in Figure 5. Generally, ASDs that displayed better solubility were found to exhibit higher dissolution compared to SD. The release from ASD8 was found to be significantly higher compared to CEF (*p* < 0.0001) as well as other batches (*p* < 0.001) of ASDs. The higher drug release from ASD8 can most likely be attributed to a loss of the crystalline nature of CEF and better wettability of the ASD. As CEF is likely to be reduced to an amorphous form in ASD, no energy would be required to break the drug crystal lattice. Thus, these ASDs generally display substantially higher apparent solubility and faster dissolution rates [22,26]. In addition, the presence of an alkalizer in ASDs modulates the microenvironment pH, preventing drug precipitation and facilitating release of CEF in challenging pH conditions. Modulation of microenvironment pH using alkalizers is employed as a promising strategy to enhance the dissolution of solid dispersions of acidic drugs using pH sensitive hydrophilic polymers [27,28]. Alkalizers such as bicarbonate and meglumine have been employed as alkalizing agents to improve the dissolution of poorly soluble drugs developed using solid dispersions made of hydrophilic polymers [24]. Considering the better solubility and dissolution of ASD8, compared to others, the batch was subjected to extensive characterization by Fourier transform infrared (FTIR), scanning electron microscopy (SEM), differential scanning calorimetry (DSC), and powder X-ray diffraction (PXRD), and eventually assessed for antimicrobial efficacy.

In order to assess possible chemical interactions between CEF and excipients used, the FTIR spectra of the CEF, PEG 6000, meglumine, physical mixture, and ASD8 were recorded. The results also ruled out the possibility of any chemical interaction between CEF and excipients during the preparation, as indicated in Figure 6. The data observed proved the chemical integrity of CEF in SD4 and ASD8 was produced by the solvent evaporation technique.

The melting point observed in the DSC corresponds to the melting point of CEF, which is reported to be at 227.59 °C. The ∆Hf value observed for CEF was found to match with that reported in the literature, confirming its crystalline nature [9]. However, the heat of fusion of the physical mixture reduced to 92.43 J/g, indicating a decrease in crystallinity to nearly 80%. The reduction in the peak intensity in the mixture suggests that ~20% of CEF is solubilized in PEG 6000 on melting. A similar drop in the peak intensity of CEF in the physical mixture with a hydrophobic carrier was found in earlier reports [9]. The DSC data infer that nearly 98% of the drug was in an amorphous form or in a molecular state as a solid–solid solution in SD4 as well as ASD8 (Figure 7). The results clearly indicated that the crystallinity of the drug was substantially reduced in these batches.

PXRD was used in conjunction with DSC to determine the solid state of the drug in the solid dispersion. The XRD spectra of the PEG 6000, meglumine, physical mixture, SD4, and ASD8 are depicted in Figure 8. The PXRD of the peaks of CEF was reported at 2θ values of 14.79°, 17.86°, 19.16°, 21.58°, and 24.5°, authenticating the drug [10]. The degree of crystallinity (DC) was calculated considering the peak at 19° 2θ value as the reference peak as it displayed the highest intensity [10]. The DC of CEF in physical mixture was found to drop to nearly 37% of the crystalline CEF. The crystallinity was found to further drop to 5% of CEF in SD4 and ASD8, indicating 95% of the drug was in the amorphous form. The DSC and PXRD studies conclusively prove that CEF was nearly reduced to amorphous state as a solid–solid solution in PEG 6000. However, no significant difference in the crystallinity was noted between SD4 and ASD8. The molecular state of drug would be one of the possible reasons for the better solubility and dissolution of the CEF from ASD8.

SEM clearly indicated the smaller particle size of ASD8 compared to CEF. The reduction in the particle size would generate a higher effective surface area that could eventually improve the solubility and dissolution of ASD8 [7].

ASD8 were found to display significantly higher (*p* < 0.001) zone of inhibition against Gram-positive (*S. aureus*) and Gram-negative (*E. coli*) species. The better antimicrobial efficacy can be attributed to the better diffusion of ASD8 compared to CEF by virtue of its higher solubility that would result in a higher thermodynamic activity. The better anti-microbial activity can improve the curing rates by avoiding development of drug-resistant strains. ASD8 have the potential to generate higher flux across membrane owing to a higher supersaturation. ASD8 have the potential to reduce the minimum inhibitory concentrations, and thus improve antimicrobial activity. Superior antimicrobial activity of solid dispersions of poorly soluble broad-spectrum antibiotic is related to the improved permeability across the bacterial cell wall [29]. The enhanced permeability can be attributed to higher thermodynamic activity as a result of better solubility. A similar improvement in the efficacy of CEF has been reported earlier by β-cyclodextrin complexes of CEF compared to drug per se. The improved solubility of the complexes was considered to be the possible reason for the better efficacy [10].

ODTs of ASD8 were developed by direct compression method using directly compressible (DC) grades of mannitol and microcrystalline cellulose as excipients. These DC materials were known to impart good flowability and compressibility of the dry mix. In addition, mannitol used as a sweetener produces a cooling effect and presents a good mouth feel. Crospovidone K30 and Croscarmellose sodium incorporated in the formulation served as super disintegrants.

The tablet formulations displayed a weight variation range from 148 ± 1.26 to 152 ± 1.8 mg. The results of the weight variation were found to meet the compendia limits [19]. The HR lower than 1.25 indicates the powder would be free-flowing, while the CI below 20% indicates good flowability [18]. The good flow properties can be attributed to the incorporation of the directly compressible materials into the formulation. By virtue of the good pre-compressional properties, the lubricated blend composed of ASD8 can be compressed without any flaws such as sticking, picking, lamination, and capping [30]. Ammonium bicarbonate was used as pore forming subliming agent into four formulations (F1, F3, F4, and F5). The subliming agent was found to render the tablets porous on vacuum drying. The bicarbonate is known to sublime at 36 °C as it decomposes to form ammonia, carbon dioxide, and water. Ammonium bicarbonate has been used as a subliming agent in development of porous orally disintegrating tablets [31,32].

Mouth-dissolving tablets having a hardness in the range of 3–4 kg were found to possess sufficient mechanical strength and at the same time enable quick wetting time and disintegration [15,33]. The absorption and wetting of the ODTs are known to determine the disintegration time. In the present work, in addition to incorporation of super disintegrants, the tablets were rendered porous by the addition of a subliming agent that eventually would be sublimed when the tablets were subjected to vacuum drying.

All the porous tablets composed of ASD8 were found to display a significantly shorter wetting time (*p* < 0.05) compared to those noted with porous tablets of CEF (batch F1). The shorter wetting time observed is probably due to rapid diffusion and quicker uptake of water into microchannels in the porous tablet. The shorter wetting time can also be ascribed to the better wettability of the hydrophilic ASD8 compared to drug per se. Higher wettability has been reported in solid dispersions composed of hydrophilic polymers [22]. The shortest wetting time (17.21 ± 1.79 s) was observed with porous tablets F5 containing CEFSD along with croscarmellose sodium (10%) and crospovidone (5%) as superdisintegrants. Wetting time is invariably going to determine the tablet disintegration and the in vitro dissolution of porous fast dissolving tablets [16].

The disintegration time (DT) noted in the present study was found to be directly related to the wetting time as the DT was found to drop with shorter wetting time. The permissible limit of DT of an ODT when determined in a USP disintegration apparatus must not exceed 30 s [33]. Generally, porous ODTs were found to display a shorter DT (27.11 ± 1.96 to 88.22 ± 13.23 s) compared to their non-porous counterparts (33.45 ± 3.65 to 98.34 ± 12.27 s). For instance, the porous tablets of F5 containing ASD8 displayed the shortest DT (27.11 ± 1.96 s), significantly shorter (*p* < 0.001) compared to the other porous ODTs developed. The possible reason could be the creation of microchannels by the subliming agent that render the tablets porous and promote water uptake, thereby substantially reducing the DT. Similar observations were made with orally disintegrating tablets composed of subliming agent [16]. In addition, the shorter DT noted with the ODTs of ASD8 can also be ascribed to the relatively hydrophilic nature of ASD8 compared to CEF that would result in better wettability [22]. Moreover, the rapid DT can also be attributed to the superdisintegrants incorporated in the tablets. The superdisintegrants croscarmellose sodium and crospovidone incorporated in the ODTs contributed to the quicker disintegration. Superdisintegrants are commonly employed to reduce DT and quicken drug release by potentially promoting water absorption by mechanisms such as swelling and wicking [16]. Croscarmellose sodium is a superdisintegrant that displays rapid water uptake and high swelling ability independent of the medium pH. Crospovidone is hydrophilic cross-linked polyvinylpyrrolidone that is commonly used as a superdisintegrant. A combination of the two superdisintegrants has been employed to improve the DT and eventually the drug release from porous tablets [34].

Generally, the drug release (68.99 ± 2.19 to 93.85 ± 2.27%) was found to be much higher for ODTs composed of ASD8 than the release from ODTs containing CEF (49.68 ± 1.23% to 62.47 ± 1.98%) per se (Figure 10). For instance, the drug release was found to significantly greater (*p* < 0.0001) for batch F5 (93.85 ± 2.27%) compared to batch F1 (62.47 ± 1.98%), indicating the effect of ASD8 on the drug release. The presence of CEF in amorphous or molecular state in the hydrophilic matrix can account for the better wetting and higher drug release from tablets composed of ASD8. The molecular state of drug in solid dispersions was considered to be one of the prime reasons for the better drug release from ODTs [15,35].

It was also noteworthy that the drug release from tablets depended on tablet porosity as well. For instance, the drug release (62.48 ± 1.98%) from a porous tablet composed of CEF (F1) was found to be significantly lower (*p* < 0.0002) compared to the non-porous counterpart F2 that displayed a release of 49.68 ± 1.23%, indicating the effect of incorporation of subliming agent. Likewise, drug release from porous tablets of ASD8 was generally found to much higher the non-porous counterparts. For instance, the drug release from porous tablets (F5) of ASD8 was found to be 93.85 ± 2.27%, significantly higher (*p* < 0.005) than non-porous tablets F8 composed of ASD8 (82.86 ± 0.96%). The higher release from the porous tablets can be ascribed to the channelizing effect of the subliming agents that promote water uptake into microchannels in porous tablets [16,31].

Normally, the tablet disintegration is the rate determining step that precedes dissolution of ODTs. More often, the disintegration of ODTs invariably determines the drug dissolution rate [20]. Therefore, the wetting and disintegration of tablets would be rate-limiting steps that affect the release, and therefore absorption, of poorly soluble drugs [36]. In the present study, a rank order correlation was found to exist between the DT and the amount dissolved at 30 min for ODTs. For instance, the porous tablets of ASD8 and F5 with shortest DT (27.11 ± 1.96 s) displayed maximum drug release (93.85 ± 2.27%). The ODT (F5) that was found to exhibit a significantly better (*p* < 0.005) drug release compared to any other ODTs of ASD8 was considered as the optimal batch. On the contrary, minimum drug release of 49.68 ± 1.23% was recorded with nonporous tablets of CEF (F2) that displayed prolonged DT (98.34 ± 12.27 s), indicating the impact of DT on drug release. The quicker drug release can be related to shorter DT due to incorporation of superdisintegrants like croscarmellose sodium and crospovidone in the ODTs. A pronounced effect of the combination of two different superdisintegrants on the drug release has been noted in earlier studies [16]. Comparison of the dissolution profiles of F5 with the constituent ASD8 indicated that the profiles were similar with F1 and F2 values of 63 and 11, respectively. These results indicate the process of direct compression did not affect the dissolution profiles of the ODTs of ASD8. In addition, it was also noted that the pH of the dissolution media following the studies with the batch F1 and F2 that were devoid of alkalizers were unaltered. However, when the pH was determined upon completion of dissolution of tablets of batch F5, the pH of the media after the studies increased from 1.2 ± 0.1 to 1.24 ± 0.2. These observations clearly elucidated the role of alkalizers in improving the dissolution by modulating the microenvironment pH. It is well documented that alkalizers are known to modulate the microenvironment pH without drastically affecting the bulk dissolution media pH [13].

The stability data revealed that there were no changes in physical characteristics, including the critical tablet properties like content uniformity, DT, friability, and in vitro dissolution profile, following the studies. From the data collected, it can be concluded that the ODT F5 was deemed to be stable when subjected to stability analysis for three months.

## 5. Conclusions

Solid dispersion of Cefdinir with the improved solubility was successfully produced by solvent evaporation technique using PEG 6000 as a carrier and meglumine as a pH modulator. Solid state characterization by DSC and XRD indicated that the drug is likely to be present in amorphous form as solid–solid solution in PEG 6000. The porous ODTs produced by direct compression followed by vacuum drying were found to display quick disintegration time and near complete release in 30 min. Thus, the ODTs are likely to deliver CEF in dissolved form, proximal to the absorption site situated in the small intestine, and therefore could improve the absorption and oral bioavailability. The ODTs composed of ASD8 are an attractive platform to resolve the solubility and dissolution issues associated with CEF in the challenging physiological pH conditions in the stomach. Thus, ODTs of CEF have the potential to effectively manage various respiratory infections and prevent the development of drug-resistant strains, thereby improving the curing rates.

## Figures and Tables

**Table 1:** Composition of SD and ASD of Cefdinir in mg.

FORMULATION	DRUG	PVP K30	PEG 6000	MEGLUMINE	SODIUM BICARBONATE
SD1	200	100	-	-	-
SD2	200	200	-	-	-
SD3	200	-	100	-	-
SD4	200	-	200	-	-
ASD1	200	100	-	-	17
ASD2	200	200	-	-	17
ASD3	200	100	-	17	-
ASD4	200	200	-	17	-
ASD5	200	-	100	-	17
ASD6	200	-	200	-	17
ASD7	200	-	100	17	-
ASD8	200	-	200	17	-

**Table 2:** Composition of rapid orodispersible tablets of CEF.

Ingredients (mg)	F1	F2	F3	F4	F5	F6	F7	F8
CEF	50	50	-	-	-	-	-	-
ASD8	-	-	100	100	100	100	100	100
Mannitol *	57	67	11	11	11	15	15	15
Microcrystalline cellulose *	3	-	-	3	9	6	3	3
Croscarmellose sodium	15	15	15	15	15	15	15	15
Crospovidone	12	15	2	5	8	12	14	16
Ammonium bicarbonate	10	-	10	10	10	-	-	-
Magnesium stearate	2	2	2	2	2	2	2	2
Talc	1	1	1	1	1	1	1	1

* Indicates directly compressible grades; The ASD contains 50 mg of CEF.

**Table 3:** Phase solubility calculation details of CEF in different solubilizer.

Solubilizer	Slope (sl)	Intercept	1 − sl	Int (1 − sl)	Ks (L·g^−1^)
PVP K30	0.0883	5.072	0.912	4.625664	0.019024
PEG 6000	0.0971	5.153	0.903	4.655868	0.020834
Soluplus^®^	0.079	4.977	0.921	4.583817	0.017235

**Table 4:** Pre-compressional properties of lubricated blend F1-F8.

Formulation Code	Bulk Density (gm/cm^3^)	Tapped Density (gm/cm^3^)	Hausner’s Ratio	Carr’s Index	Angle of Repose
F1	0.553 ± 0.03	0.63 ± 0.03	1.13 ± 0.04	12.02 ± 0.89	21.65 ± 2.05
F2	0.564 ± 0.05	0.65 ± 0.02	1.15 ± 0.03	14.28 ± 1.12	20.21 ± 2.26
F3	0.562 ± 0.006	0.64 ± 0.01	1.08 ± 0.02	7.79 ± 1.65	21.56 ± 3.17
F4	0.503 ± 0.015	0.60 ± 0.01	1.06 ± 0.01	7.57 ± 0.76	20.18 ± 2.21
F5	0.543 ± 0.015	0.59 ± 0.015	1.05 ± 0.02	7.93 ± 0.54	19.92 ± 2.05
F6	0.551 ± 0.01	0.62 ± 0.01	1.11 ± 0.07	7.98 ± 0.32	21.23 ± 1.24
F7	0.563 ± 0.005	0.58 ± 0.01	1.07 ± 0.01	7.95 ± 0.23	21.70 ± 1.55
F8	0.552 ± 0.01	0.61 ± 0.01	1.09 ± 0.015	8.02 ± 0.28	21.62 ± 2.03

**Table 5:** Post-compressional parameters of prepared orodispersible tablets.

Formulation Code	Weight Variation (mg)	Hardness (Kg)	Thickness (mm)	%Friability	%Content Uniformity	Wetting Time (s)	Disintegration Time (s)	%Water Absorption Ratio
F1	150 ± 1.73	3.41 ± 0.25	2.75 ± 0.04	0.25 ± 0.04	89.12 ± 1.23	22.12 ± 1.25	45.46 ± 3.23	24.2 ± 1.4
F2	150 ± 1.32	3.37 ± 0.28	2.79 ± 0.03	0.28 ± 0.07	88.32 ± 1.24	23.22 ± 1.54	40.89 ± 2.27	26.4 ± 1.5
F3	151 ± 1.67	3.50 ± 0.25	3.02 ± 0.02	0.26 ± 0.05	88.82 ± 1.14	18.14 ± 1.31	32.41 ± 4.06	20.1 ± 1.37
F4	149 ± 1.29	3.22 ± 0.24	3.01 ± 0.01	0.29 ± 0.09	93.34 ± 1.24	19.11 ± 1.29	30.25 ± 1.15	18.9 ± 2.33
F5	150 ± 1.23	3.12 ± 0.28	2.99 ± 0.03	0.25 ± 0.04	96.46 ± 0.84	17.21 ± 1.79	27.11 ± 1.06	19.1 ± 2.24
F6	148 ± 1.26	3.32 ± 0.21	3.05 ± 0.06	0.26 ± 0.05	90.14 ± 1.05	20.11 ± 2.58	34.45 ± 2.06	20.51 ± 1.26
F7	152 ± 1.8	3.46 ± 0.14	3.01 ± 0.04	0.27 ± 0.06	90.12 ± 1.12	20.42 ± 1.89	33.45 ± 3.65	19.72 ± 1.76
F8	147 ± 1.3	3.32 ± 0.18	3.02 ± 0.03	0.28 ± 0.07	91.10 ± 1.15	21.32 ± 1.33	37.34 ± 8.23	22.11 ± 1.15

**Table 6:** Results of stability studies of the orodispersible tablets (batch F5).

Evaluation Parameters	Initial	25 ± 2 °C/60 ± 5%RH			40 ± 2 °C/75 ± 5%RH		
	0-Month	1-Month	2-Month	3-Month	1-Month	2-Month	3-Month
Average weight (mg)	150 ± 1.23	150.01 ± 1.21	150.02 ± 1.24	150.02 ± 1.26	149.8 ± 1.27	149.6 ± 1.46	149.5 ± 1.16
Hardness (kg/cm^2^)	3.12 ± 0.28	3.1 ± 0.23	3.1 ± 0.22	3.1 ± 0.27	3.02 ± 0.25	3.05 ± 0.28	3.06 ± 0.22
Friability (%)	0.25 ± 0.04	0.25 ± 0.02	0.24 ± 0.06	0.24 ± 0.03	0.24 ± 0.03	0.24 ± 0.06	0.23 ± 0.05
Drug content (%)	96.46 ± 0.84	96.11 ± 0.86	96.10 ± 0.83	96.09 ± 0.87	95.38 ± 0.79	95.45 ± 0.97	95.52 ± 0.89
Disintegration time (s)	27.11 ± 1.06	26 ± 1.87	26 ± 1.95	25 ± 1.98	27 ± 1.94	28 ± 1.95	29 ± 1.97
%Cumulative drug release (30 min)	93.91 ± 0.12	93.34 ± 0.28	93.46 ± 0.34	93.54 ± 0.28	92.48 ± 0.15	92.75 ± 0.12	92.98 ± 0.05

## Data Availability

All data analyzed or generated during this study are included in this article.
